# (η^6^-Benzene)(carbonato-κ^2^ *O*,*O*′)[di­cyclohex­yl(naphthalen-1-ylmeth­yl)phosphane-κ*P*]ruthenium(II) chloro­form tris­olvate

**DOI:** 10.1107/S1600536814014081

**Published:** 2014-06-21

**Authors:** Saravanan Gowrisankar, Helfried Neumann, Anke Spannenberg, Matthias Beller

**Affiliations:** a Division of Organic Chemistry, Institute of Chemical and Engineering Sciences, 8 Biomedical grove, Neuros, #07-01, 138665, Singapore; b Leibniz-Institut für Katalyse e. V. an der Universität Rostock, Albert-Einstein-Strasse 29a, 18059 Rostock, Germany

**Keywords:** crystal structure

## Abstract

The title compound, [Ru(CO_3_)(η^6^-C_6_H_6_){(C_6_H_11_)_2_P(CH_2_C_10_H_7_)}]·3CHCl_3_, was synthesized by carbonation of [RuCl_2_(η^6^-C_6_H_6_){(C_6_H_11_)_2_P(CH_2_C_10_H_7_)}] with NaHCO_3_ in methanol at room temperature. The Ru^II^ atom is surrounded by a benzene ligand, a chelating carbonate group and a phosphane ligand in a piano-stool configuration. The crystal packing is consolidated by C—H⋯O and C—H⋯Cl hydrogen-bonding inter­actions between adjacent metal complexes and between the complexes and the solvent mol­ecules. The asymmetric unit contains one metal complex and three chloro­form solvent mol­ecules of which only one was modelled. The estimated diffraction contributions of the other two strongly disordered chloro­form solvent mol­ecules were substracted from the observed diffraction data using the SQUEEZE procedure in *PLATON* [Spek (2009 ▶). *Acta Cryst.* D**65**, 148–155].

## Related literature   

For crystal structures of related carbonatophosphane ruthenium(II) complexes, see: Allen *et al.* (2009 ▶); Blosser *et al.* (2004 ▶); Davies *et al.* (2013 ▶); Dell’Amico *et al.* (2000 ▶); Demerseman *et al.* (2006 ▶); Drake *et al.* (2013 ▶). The starting complex [RuCl_2_(η^6^-C_6_H_6_)(C_6_H_11_)_2_PCH_2_C_10_H_7_)] was described by Gowrisankar *et al.* (2014 ▶).
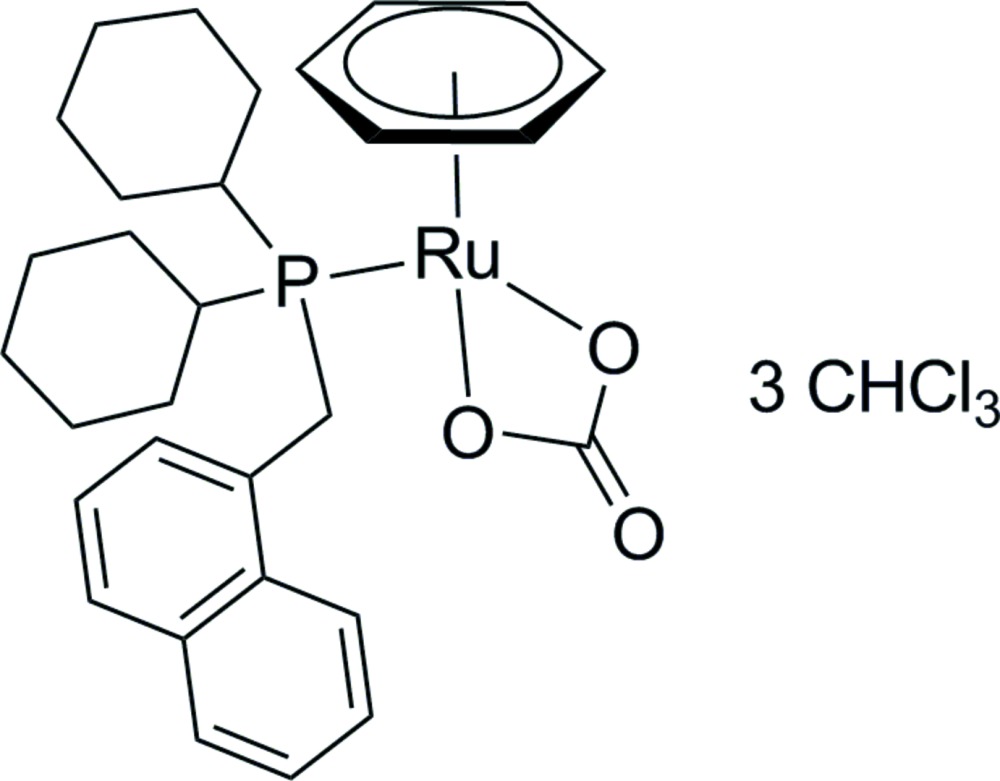


## Experimental   

### 

#### Crystal data  

[Ru(CO_3_)(C_6_H_6_)(C_23_H_31_P)]·3CHCl_3_
*M* *_r_* = 935.74
Orthorhombic, 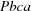

*a* = 22.1730 (4) Å
*b* = 15.1385 (3) Å
*c* = 23.6954 (5) Å
*V* = 7953.7 (3) Å^3^
*Z* = 8
Cu *K*α radiation
μ = 9.40 mm^−1^
*T* = 150 K
0.64 × 0.08 × 0.05 mm

#### Data collection  

Bruker Kappa APEXII DUO diffractometer
Absorption correction: multi-scan (*SADABS*; Bruker, 2008 ▶) *T*_min_ = 0.065, *T*_max_ = 0.651
68879 measured reflections
6998 independent reflections
5815 reflections with *I* > 2σ(*I*)
*R*_int_ = 0.062

#### Refinement  

*R*[*F* ^2^ > 2σ(*F* ^2^)] = 0.041
*wR*(*F* ^2^) = 0.110
*S* = 1.07
6998 reflections
352 parameters
H-atom parameters constrained
Δρ_max_ = 1.17 e Å^−3^
Δρ_min_ = −0.79 e Å^−3^

### 

Data collection: *APEX2* (Bruker, 2012 ▶); cell refinement: *SAINT* (Bruker, 2009 ▶); data reduction: *SAINT*; program(s) used to solve structure: *SHELXS97* (Sheldrick, 2008 ▶); program(s) used to refine structure: *SHELXL2014* (Sheldrick, 2008 ▶); molecular graphics: *XP* in *SHELXTL* (Sheldrick, 2008 ▶); software used to prepare material for publication: *SHELXL2014* and *PLATON* (Spek, 2009 ▶).

## Supplementary Material

Crystal structure: contains datablock(s) I, Global. DOI: 10.1107/S1600536814014081/wm5028sup1.cif

Structure factors: contains datablock(s) I. DOI: 10.1107/S1600536814014081/wm5028Isup2.hkl

CCDC reference: 1008559

Additional supporting information:  crystallographic information; 3D view; checkCIF report

## Figures and Tables

**Table 1:** Hydrogen-bond geometry (Å, °)

*D*—H⋯*A*	*D*—H	H⋯*A*	*D*⋯*A*	*D*—H⋯*A*
C6—H6⋯O3^i^	0.95	2.51	3.179 (5)	127
C29—H29*A*⋯Cl3^ii^	0.99	2.68	3.457 (6)	135
C31—H31⋯O3^iii^	1.00	2.06	3.004 (5)	156

Symmetry codes: (i) 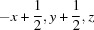; (ii) 
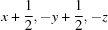
; (iii) 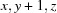.

**Fig. 1.**
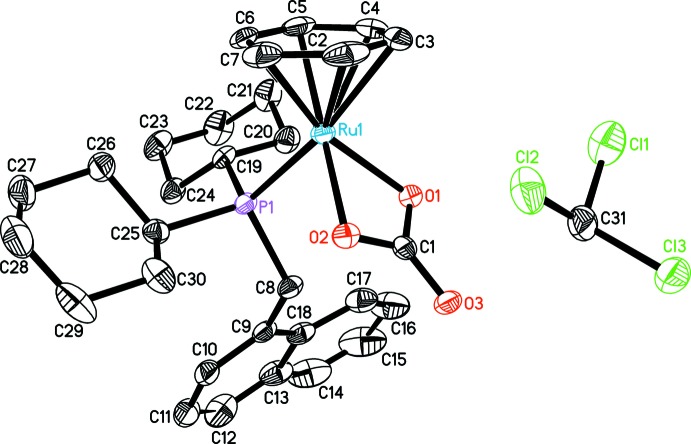
The molecular structure of the title compound with atoms at the 30% probability level for the displacement ellipsoids. Hydrogen atoms are omitted for clarity.

**Fig. 2.**
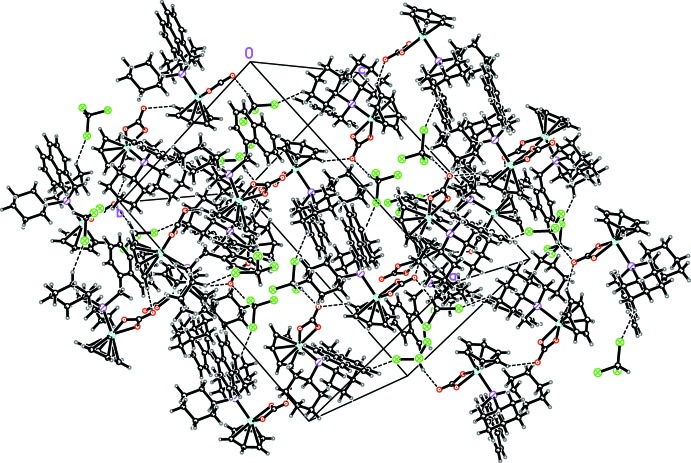
A packing diagram of the title compound showing hydrogen bonds as dashed lines.

## supplementary crystallographic information

### S1. Comment

The title compound was prepared in a two step synthesis: In the presence of 60 bar hydrogen, the benzene ruthenium dichloride dimer and the dicyclohexyl(1-naphthoyl)phosphane ligand react to give the [RuCl_2_(η^6^-C_6_H_6_){(C_6_H_11_)_2_PCH_2_C_10_H_7_)}] complex in 80% yield. Here, the carbonyl group of the ligand is reduced to a methylen unit (Gowrisankar *et al.*, 2014). In the second step the reduced complex was carbonated at room temperature in methanol with 10 equivalent of NaHCO_3_ to yield the title compound, [Ru(CO_3_)(η^6^-C_6_H_6_){(C_6_H_11_)_2_PCH_2_C_10_H_7_)}], as a chloroform solvate after recrystallization from a CHCl_3_/heptane mixture. Related carbonatophosphane ruthenium complexes are known from the literature (Allen *et al.*, 2009; Blosser *et al.*, 2004; Davies *et al.*, 2013; Dell'Amico *et al.*, 2000; Demerseman *et al.*, 2006; Drake *et al.*, 2013).

The asymmetric unit contains one complex and one chloroform solvent molecule (Fig. 1). Contributions of two further strongly disordered solvent molecules (chloroform) were removed from the diffraction data with the SQUEEZE option in *PLATON* (Spek, 2009). The Ru^II^ atom is surrounded by a benzene ligand, a chelating carbonate group and a phosphane ligand (C_6_H_11_)_2_P(CH_2_C_10_H_7_) in a piano-stool geometry. The phosphane ligand is linked through its P atom with a Ru—P bond length of 2.3705 (8) Å; both cyclohexyl rings at the P atom adopt a chair conformation. The molecular structure shows a planar arrangement of the Ru(CO_3_) fragment (mean deviation of the best plane defined by Ru1, O1, C1, O2, O3 is 0.036 Å). As expected, the exocyclic C—O bond in the Ru(CO_3_) unit is with 1.242 (4) Å significantly shorter than the two endocyclic C—O bonds (C1—O1 = 1.326 (4) and C1—O2 = 1.309 (4) Å). The complex molecules as well as complex and solvent molecules are linked by C—H···Cl and C—H···O hydrogen bonds (Fig. 2).

The ^31^P-NMR spectrum of the title complex shows a singulett at 42.5 p.p.m., whereas the reduced complex [RuCl_2_(η^6^-C_6_H_6_){(C_6_H_11_)_2_PCH_2_C_10_H_7_)}] exhibits an upfield shift to 39.1 p.p.m..

### S2. Experimental

At room temperature, a mixture consisting of [RuCl_2_(η^6^-C_6_H_6_){(C_6_H_11_)_2_P(1-methylnaphthyl)}] (20 mg, 0.034 mmol), NaHCO_3_(63 mg, 0.34 mmol) and methanol (5 ml) was stirred under argon in a Schlenk tube. The orange suspension changed to a yellow solution. The reaction was completed within 10 min and the solution was filtered over celite. The solvent was removed *in vacuo* and 18 mg (94%) of a yellow solid was obtained. Crystals suitable for X-ray analysis were grown from a CHCl_3_/heptane mixture at 245 K. ^1^H NMR (300 MHz, CDCl_3_) δ 8.18 (d, *J* = 8.6 Hz, 1H, naphthyl), 7.88–7.75 (m, 2H, naphthyl), 7.63–7.30 (m, 3H, naphthyl), 7.33 (m, 1H, naphthyl), 5.07 (s, 6H, benzene), 3.55 (d, *J* = 10.2 Hz, 2H, CH_2_), 2.14–1.95 (m, 4H, Cy), 1.95–1.79 (m, 4H, Cy), 1.79–1.65 (m, 4H, Cy), 1.67–1.47 (m, 2H, Cy), 1.32–1.01 (m, 8H, Cy). ^31^P{^1^H} NMR (121 MHz, CDCl_3_): δ 42.5.

### S3. Refinement

H atoms were placed in idealized positions with *d*(C—H) = 0.95 - 1.00 Å (CH), 0.99 Å (CH_2_) and refined using a riding model with *U*_iso_(H) fixed at 1.2*U*_eq_(C). Contributions of further disordered solvent molecules were removed from the diffraction data with *PLATON* / SQUEEZE (Spek, 2009). SQUEEZE estimated the electron count in the void volume of 1149 Å^3^ to be 501; two voids are given. The highest peak in the final difference Fourier map is located 0.86 Å from Ru1 and the deepest hole 0.75 Å from Cl3.

### Crystal data

**Table:**

[Ru(CO_3_)(C_6_H_6_)(C_23_H_31_P)]·3CHCl_3_	*D*_x_ = 1.563 Mg m^−^^3^
*M**_r_* = 935.74	Cu *K*α radiation, λ = 1.54178 Å
Orthorhombic, *P**b**c**a*	Cell parameters from 9902 reflections
*a* = 22.1730 (4) Å	θ = 3.7–66.2°
*b* = 15.1385 (3) Å	µ = 9.40 mm^−^^1^
*c* = 23.6954 (5) Å	*T* = 150 K
*V* = 7953.7 (3) Å^3^	Needle, yellow
*Z* = 8	0.64 × 0.08 × 0.05 mm
*F*(000) = 3792	

### Data collection

**Table:**

Bruker Kappa APEXII DUO diffractometer	6998 independent reflections
Radiation source: microfocus	5815 reflections with *I* > 2σ(*I*)
Multilayer monochromator	*R*_int_ = 0.062
Detector resolution: 8.3333 pixels mm^-1^	θ_max_ = 66.6°, θ_min_ = 3.7°
ω and φ scans	*h* = −26→25
Absorption correction: multi-scan (*SADABS*; Bruker, 2008)	*k* = −17→17
*T*_min_ = 0.065, *T*_max_ = 0.651	*l* = −28→27
68879 measured reflections	

### Refinement

**Table:**

Refinement on *F*^2^	0 restraints
Least-squares matrix: full	Hydrogen site location: inferred from neighbouring sites
*R*[*F*^2^ > 2σ(*F*^2^)] = 0.041	H-atom parameters constrained
*wR*(*F*^2^) = 0.110	*w* = 1/[σ^2^(*F*_o_^2^) + (0.050*P*)^2^ + 8.4698*P*] where *P* = (*F*_o_^2^ + 2*F*_c_^2^)/3
*S* = 1.07	(Δ/σ)_max_ < 0.001
6998 reflections	Δρ_max_ = 1.17 e Å^−^^3^
352 parameters	Δρ_min_ = −0.79 e Å^−^^3^

### Special details

**Table:**

Geometry. All e.s.d.'s (except the e.s.d. in the dihedral angle between two l.s. planes) are estimated using the full covariance matrix. The cell e.s.d.'s are taken into account individually in the estimation of e.s.d.'s in distances, angles and torsion angles; correlations between e.s.d.'s in cell parameters are only used when they are defined by crystal symmetry. An approximate (isotropic) treatment of cell e.s.d.'s is used for estimating e.s.d.'s involving l.s. planes.

### Fractional atomic coordinates and isotropic or equivalent isotropic displacement parameters (Å^2^)

**Table:**

	*x*	*y*	*z*	*U*_iso_*/*U*_eq_	
C31	0.03361 (18)	0.8411 (3)	0.0554 (2)	0.0638 (12)	
H31	0.0732	0.8184	0.0695	0.077*	
Cl1	0.00779 (7)	0.92264 (10)	0.10140 (6)	0.0870 (4)	
Cl2	0.04409 (5)	0.88587 (10)	−0.01171 (6)	0.0783 (4)	
Cl3	−0.01875 (5)	0.75354 (8)	0.05459 (7)	0.0812 (4)	
C1	0.18339 (13)	−0.1129 (2)	0.06775 (15)	0.0346 (7)	
C2	0.1466 (2)	0.1023 (3)	−0.0075 (2)	0.0668 (14)	
H2	0.1235	0.0740	−0.0361	0.080*	
C3	0.12336 (17)	0.1126 (3)	0.0464 (2)	0.0598 (12)	
H3	0.0836	0.0931	0.0544	0.072*	
C4	0.15773 (19)	0.1512 (2)	0.0888 (2)	0.0572 (11)	
H4	0.1424	0.1537	0.1262	0.069*	
C5	0.21390 (18)	0.1860 (2)	0.07708 (19)	0.0509 (9)	
H5	0.2362	0.2152	0.1057	0.061*	
C6	0.23753 (16)	0.1778 (2)	0.02288 (19)	0.0489 (9)	
H6	0.2761	0.2022	0.0150	0.059*	
C7	0.2066 (2)	0.1351 (3)	−0.01994 (19)	0.0580 (11)	
H7	0.2241	0.1276	−0.0562	0.070*	
C8	0.31201 (14)	−0.1025 (2)	0.11038 (15)	0.0371 (7)	
H8A	0.2770	−0.1058	0.1362	0.045*	
H8B	0.3036	−0.1438	0.0789	0.045*	
C9	0.36532 (14)	−0.1397 (2)	0.14218 (16)	0.0387 (8)	
C10	0.41559 (16)	−0.1674 (3)	0.1131 (2)	0.0533 (10)	
H10	0.4172	−0.1610	0.0733	0.064*	
C11	0.46469 (19)	−0.2052 (3)	0.1421 (3)	0.0810 (18)	
H11	0.4989	−0.2247	0.1215	0.097*	
C12	0.4639 (2)	−0.2142 (4)	0.1988 (3)	0.0815 (17)	
H12	0.4980	−0.2387	0.2174	0.098*	
C13	0.4139 (2)	−0.1880 (3)	0.2304 (2)	0.0621 (12)	
C14	0.4120 (3)	−0.1993 (4)	0.2895 (2)	0.0862 (18)	
H14	0.4468	−0.2210	0.3084	0.103*	
C15	0.3619 (4)	−0.1801 (4)	0.3205 (3)	0.101 (2)	
H15	0.3615	−0.1887	0.3602	0.121*	
C16	0.3109 (3)	−0.1471 (4)	0.2924 (2)	0.0869 (17)	
H16	0.2757	−0.1337	0.3136	0.104*	
C17	0.3106 (2)	−0.1339 (3)	0.23569 (19)	0.0598 (11)	
H17	0.2751	−0.1120	0.2182	0.072*	
C18	0.36244 (16)	−0.1520 (2)	0.20162 (17)	0.0443 (8)	
C19	0.34391 (14)	0.0887 (2)	0.13099 (14)	0.0356 (7)	
H19	0.3404	0.1482	0.1129	0.043*	
C20	0.30672 (17)	0.0935 (2)	0.18484 (16)	0.0461 (8)	
H20A	0.3111	0.0376	0.2062	0.055*	
H20B	0.2636	0.1010	0.1752	0.055*	
C21	0.3276 (2)	0.1705 (3)	0.22123 (19)	0.0596 (11)	
H21A	0.3037	0.1721	0.2565	0.072*	
H21B	0.3208	0.2267	0.2008	0.072*	
C22	0.3943 (2)	0.1608 (4)	0.2353 (2)	0.0681 (13)	
H22A	0.4077	0.2126	0.2575	0.082*	
H22B	0.4003	0.1074	0.2587	0.082*	
C23	0.43187 (18)	0.1536 (3)	0.18300 (19)	0.0576 (11)	
H23A	0.4746	0.1445	0.1937	0.069*	
H23B	0.4292	0.2096	0.1615	0.069*	
C24	0.41101 (15)	0.0771 (2)	0.14555 (17)	0.0450 (8)	
H24A	0.4170	0.0204	0.1656	0.054*	
H24B	0.4352	0.0758	0.1104	0.054*	
C25	0.36856 (15)	0.0046 (2)	0.02310 (15)	0.0390 (7)	
H25	0.4062	−0.0206	0.0399	0.047*	
C26	0.38481 (18)	0.0969 (3)	−0.00015 (18)	0.0537 (10)	
H26A	0.3488	0.1236	−0.0180	0.064*	
H26B	0.3975	0.1357	0.0313	0.064*	
C27	0.4356 (2)	0.0903 (4)	−0.0434 (2)	0.0668 (13)	
H27A	0.4448	0.1499	−0.0584	0.080*	
H27B	0.4724	0.0674	−0.0248	0.080*	
C28	0.4183 (2)	0.0297 (4)	−0.0917 (2)	0.0828 (17)	
H28A	0.3843	0.0559	−0.1129	0.099*	
H28B	0.4529	0.0236	−0.1178	0.099*	
C29	0.4004 (2)	−0.0598 (4)	−0.0700 (2)	0.0771 (16)	
H29A	0.4363	−0.0890	−0.0536	0.092*	
H29B	0.3865	−0.0963	−0.1022	0.092*	
C30	0.35006 (18)	−0.0565 (3)	−0.02506 (18)	0.0586 (11)	
H30A	0.3123	−0.0347	−0.0424	0.070*	
H30B	0.3426	−0.1166	−0.0102	0.070*	
O1	0.18027 (9)	−0.05130 (14)	0.10738 (10)	0.0363 (5)	
O2	0.20684 (9)	−0.08313 (15)	0.02076 (10)	0.0365 (5)	
O3	0.16578 (10)	−0.19007 (15)	0.07473 (12)	0.0455 (6)	
P1	0.31196 (3)	0.00947 (5)	0.08005 (4)	0.03076 (17)	
Ru1	0.21220 (2)	0.04581 (2)	0.05258 (2)	0.03286 (10)	

### Atomic displacement parameters (Å^2^)

**Table:**

	*U*^11^	*U*^22^	*U*^33^	*U*^12^	*U*^13^	*U*^23^
C31	0.040 (2)	0.064 (3)	0.088 (3)	−0.0052 (19)	−0.009 (2)	0.016 (2)
Cl1	0.0890 (9)	0.0875 (9)	0.0845 (9)	−0.0228 (7)	0.0002 (7)	−0.0021 (7)
Cl2	0.0510 (6)	0.1074 (10)	0.0766 (8)	−0.0010 (6)	0.0000 (5)	0.0143 (7)
Cl3	0.0556 (6)	0.0563 (6)	0.1317 (12)	−0.0060 (5)	−0.0115 (7)	0.0108 (7)
C1	0.0250 (14)	0.0301 (17)	0.049 (2)	0.0032 (12)	−0.0052 (13)	0.0029 (14)
C2	0.068 (3)	0.038 (2)	0.094 (4)	0.0077 (19)	−0.050 (3)	0.008 (2)
C3	0.0327 (18)	0.037 (2)	0.110 (4)	0.0105 (15)	−0.002 (2)	0.016 (2)
C4	0.059 (2)	0.0327 (19)	0.080 (3)	0.0206 (17)	0.011 (2)	0.0012 (19)
C5	0.063 (2)	0.0221 (16)	0.068 (3)	0.0063 (15)	−0.007 (2)	−0.0026 (16)
C6	0.0422 (18)	0.0268 (17)	0.078 (3)	0.0011 (14)	−0.0052 (19)	0.0159 (17)
C7	0.083 (3)	0.039 (2)	0.052 (2)	0.0145 (19)	−0.008 (2)	0.0156 (18)
C8	0.0340 (16)	0.0285 (15)	0.049 (2)	−0.0033 (13)	−0.0107 (14)	0.0073 (14)
C9	0.0334 (16)	0.0254 (15)	0.057 (2)	−0.0059 (12)	−0.0077 (15)	0.0074 (14)
C10	0.043 (2)	0.0402 (19)	0.076 (3)	0.0020 (16)	0.0048 (19)	0.0169 (19)
C11	0.038 (2)	0.050 (2)	0.154 (6)	0.0098 (18)	0.013 (3)	0.039 (3)
C12	0.046 (2)	0.074 (3)	0.125 (5)	0.000 (2)	−0.032 (3)	0.047 (3)
C13	0.064 (3)	0.046 (2)	0.077 (3)	−0.0015 (19)	−0.033 (2)	0.009 (2)
C14	0.121 (5)	0.062 (3)	0.075 (4)	0.013 (3)	−0.055 (4)	0.006 (3)
C15	0.181 (7)	0.065 (3)	0.058 (3)	0.030 (4)	−0.031 (4)	0.002 (3)
C16	0.138 (5)	0.065 (3)	0.058 (3)	0.028 (3)	0.009 (3)	0.008 (2)
C17	0.075 (3)	0.046 (2)	0.059 (3)	0.012 (2)	−0.005 (2)	0.0082 (19)
C18	0.0495 (19)	0.0279 (16)	0.055 (2)	−0.0054 (14)	−0.0157 (17)	0.0057 (15)
C19	0.0371 (16)	0.0287 (15)	0.0411 (18)	−0.0097 (13)	−0.0055 (14)	0.0017 (14)
C20	0.0454 (18)	0.044 (2)	0.049 (2)	−0.0112 (16)	−0.0032 (16)	−0.0035 (17)
C21	0.066 (3)	0.061 (3)	0.053 (2)	−0.015 (2)	0.003 (2)	−0.016 (2)
C22	0.069 (3)	0.077 (3)	0.057 (3)	−0.029 (2)	−0.015 (2)	−0.012 (2)
C23	0.052 (2)	0.054 (2)	0.066 (3)	−0.0213 (18)	−0.019 (2)	0.003 (2)
C24	0.0366 (17)	0.0430 (19)	0.056 (2)	−0.0071 (15)	−0.0097 (16)	0.0043 (16)
C25	0.0353 (16)	0.0423 (18)	0.0395 (18)	0.0049 (14)	−0.0014 (14)	0.0034 (15)
C26	0.053 (2)	0.054 (2)	0.054 (2)	0.0086 (18)	0.0118 (18)	0.0227 (19)
C27	0.061 (3)	0.077 (3)	0.062 (3)	0.016 (2)	0.020 (2)	0.025 (2)
C28	0.064 (3)	0.118 (5)	0.067 (3)	0.037 (3)	0.020 (2)	0.025 (3)
C29	0.056 (3)	0.108 (4)	0.067 (3)	0.024 (3)	0.001 (2)	−0.030 (3)
C30	0.048 (2)	0.081 (3)	0.047 (2)	0.014 (2)	−0.0018 (18)	−0.016 (2)
O1	0.0323 (11)	0.0287 (11)	0.0479 (14)	−0.0004 (8)	0.0041 (10)	0.0046 (10)
O2	0.0351 (11)	0.0357 (12)	0.0387 (13)	0.0009 (9)	−0.0075 (9)	−0.0021 (10)
O3	0.0351 (11)	0.0276 (12)	0.0739 (18)	−0.0011 (9)	−0.0048 (12)	0.0023 (12)
P1	0.0283 (4)	0.0257 (4)	0.0383 (4)	−0.0019 (3)	−0.0040 (3)	0.0038 (3)
Ru1	0.02876 (14)	0.02455 (14)	0.04527 (16)	0.00038 (8)	−0.00458 (10)	0.00306 (10)

### Geometric parameters (Å, º)

**Table:**

C31—Cl2	1.744 (5)	C16—C17	1.359 (7)
C31—Cl1	1.743 (5)	C16—H16	0.9500
C31—Cl3	1.762 (4)	C17—C18	1.431 (6)
C31—H31	1.0000	C17—H17	0.9500
C1—O3	1.242 (4)	C19—C20	1.521 (5)
C1—O2	1.309 (4)	C19—C24	1.537 (4)
C1—O1	1.326 (4)	C19—P1	1.844 (3)
C1—Ru1	2.513 (3)	C19—H19	1.0000
C2—C3	1.386 (7)	C20—C21	1.522 (5)
C2—C7	1.451 (7)	C20—H20A	0.9900
C2—Ru1	2.209 (4)	C20—H20B	0.9900
C2—H2	0.9500	C21—C22	1.524 (6)
C3—C4	1.390 (7)	C21—H21A	0.9900
C3—Ru1	2.219 (4)	C21—H21B	0.9900
C3—H3	0.9500	C22—C23	1.497 (6)
C4—C5	1.380 (6)	C22—H22A	0.9900
C4—Ru1	2.177 (4)	C22—H22B	0.9900
C4—H4	0.9500	C23—C24	1.531 (5)
C5—C6	1.393 (6)	C23—H23A	0.9900
C5—Ru1	2.200 (4)	C23—H23B	0.9900
C5—H5	0.9500	C24—H24A	0.9900
C6—C7	1.384 (6)	C24—H24B	0.9900
C6—Ru1	2.192 (3)	C25—C30	1.525 (5)
C6—H6	0.9500	C25—C26	1.545 (5)
C7—Ru1	2.190 (4)	C25—P1	1.844 (3)
C7—H7	0.9500	C25—H25	1.0000
C8—C9	1.511 (4)	C26—C27	1.526 (5)
C8—P1	1.841 (3)	C26—H26A	0.9900
C8—H8A	0.9900	C26—H26B	0.9900
C8—H8B	0.9900	C27—C28	1.516 (8)
C9—C10	1.376 (5)	C27—H27A	0.9900
C9—C18	1.422 (5)	C27—H27B	0.9900
C10—C11	1.409 (6)	C28—C29	1.502 (8)
C10—H10	0.9500	C28—H28A	0.9900
C11—C12	1.349 (8)	C28—H28B	0.9900
C11—H11	0.9500	C29—C30	1.544 (6)
C12—C13	1.397 (8)	C29—H29A	0.9900
C12—H12	0.9500	C29—H29B	0.9900
C13—C14	1.412 (8)	C30—H30A	0.9900
C13—C18	1.437 (5)	C30—H30B	0.9900
C14—C15	1.363 (9)	O1—Ru1	2.085 (2)
C14—H14	0.9500	O2—Ru1	2.096 (2)
C15—C16	1.404 (9)	P1—Ru1	2.3705 (8)
C15—H15	0.9500		
			
Cl2—C31—Cl1	109.8 (3)	C23—C22—H22B	109.3
Cl2—C31—Cl3	111.7 (3)	C21—C22—H22B	109.3
Cl1—C31—Cl3	108.8 (2)	H22A—C22—H22B	108.0
Cl2—C31—H31	108.8	C22—C23—C24	111.5 (3)
Cl1—C31—H31	108.8	C22—C23—H23A	109.3
Cl3—C31—H31	108.8	C24—C23—H23A	109.3
O3—C1—O2	124.2 (3)	C22—C23—H23B	109.3
O3—C1—O1	123.4 (3)	C24—C23—H23B	109.3
O2—C1—O1	112.4 (3)	H23A—C23—H23B	108.0
O3—C1—Ru1	176.4 (2)	C23—C24—C19	109.6 (3)
O2—C1—Ru1	56.47 (15)	C23—C24—H24A	109.8
O1—C1—Ru1	56.03 (15)	C19—C24—H24A	109.8
C3—C2—C7	119.3 (4)	C23—C24—H24B	109.8
C3—C2—Ru1	72.2 (2)	C19—C24—H24B	109.8
C7—C2—Ru1	70.1 (2)	H24A—C24—H24B	108.2
C3—C2—H2	120.4	C30—C25—C26	110.1 (3)
C7—C2—H2	120.4	C30—C25—P1	112.9 (3)
Ru1—C2—H2	129.8	C26—C25—P1	112.5 (3)
C2—C3—C4	120.7 (4)	C30—C25—H25	107.0
C2—C3—Ru1	71.3 (2)	C26—C25—H25	107.0
C4—C3—Ru1	69.9 (2)	P1—C25—H25	107.0
C2—C3—H3	119.7	C27—C26—C25	110.6 (3)
C4—C3—H3	119.7	C27—C26—H26A	109.5
Ru1—C3—H3	132.0	C25—C26—H26A	109.5
C5—C4—C3	120.7 (4)	C27—C26—H26B	109.5
C5—C4—Ru1	72.5 (2)	C25—C26—H26B	109.5
C3—C4—Ru1	73.2 (2)	H26A—C26—H26B	108.1
C5—C4—H4	119.7	C28—C27—C26	111.1 (4)
C3—C4—H4	119.7	C28—C27—H27A	109.4
Ru1—C4—H4	126.5	C26—C27—H27A	109.4
C4—C5—C6	119.4 (4)	C28—C27—H27B	109.4
C4—C5—Ru1	70.7 (2)	C26—C27—H27B	109.4
C6—C5—Ru1	71.2 (2)	H27A—C27—H27B	108.0
C4—C5—H5	120.3	C29—C28—C27	110.8 (4)
C6—C5—H5	120.3	C29—C28—H28A	109.5
Ru1—C5—H5	130.3	C27—C28—H28A	109.5
C7—C6—C5	122.1 (4)	C29—C28—H28B	109.5
C7—C6—Ru1	71.5 (2)	C27—C28—H28B	109.5
C5—C6—Ru1	71.9 (2)	H28A—C28—H28B	108.1
C7—C6—H6	119.0	C28—C29—C30	113.4 (4)
C5—C6—H6	119.0	C28—C29—H29A	108.9
Ru1—C6—H6	130.4	C30—C29—H29A	108.9
C6—C7—C2	117.8 (4)	C28—C29—H29B	108.9
C6—C7—Ru1	71.6 (2)	C30—C29—H29B	108.9
C2—C7—Ru1	71.4 (2)	H29A—C29—H29B	107.7
C6—C7—H7	121.1	C25—C30—C29	110.0 (4)
C2—C7—H7	121.1	C25—C30—H30A	109.7
Ru1—C7—H7	127.8	C29—C30—H30A	109.7
C9—C8—P1	122.6 (2)	C25—C30—H30B	109.7
C9—C8—H8A	106.7	C29—C30—H30B	109.7
P1—C8—H8A	106.7	H30A—C30—H30B	108.2
C9—C8—H8B	106.7	C1—O1—Ru1	92.15 (19)
P1—C8—H8B	106.7	C1—O2—Ru1	92.16 (19)
H8A—C8—H8B	106.6	C8—P1—C19	110.09 (15)
C10—C9—C18	119.5 (3)	C8—P1—C25	104.37 (16)
C10—C9—C8	119.9 (4)	C19—P1—C25	104.11 (15)
C18—C9—C8	120.5 (3)	C8—P1—Ru1	108.75 (10)
C9—C10—C11	120.4 (5)	C19—P1—Ru1	112.78 (11)
C9—C10—H10	119.8	C25—P1—Ru1	116.32 (11)
C11—C10—H10	119.8	O1—Ru1—O2	63.14 (9)
C12—C11—C10	121.1 (5)	O1—Ru1—C4	94.77 (13)
C12—C11—H11	119.4	O2—Ru1—C4	142.45 (13)
C10—C11—H11	119.4	O1—Ru1—C7	154.77 (14)
C11—C12—C13	121.0 (4)	O2—Ru1—C7	106.83 (13)
C11—C12—H12	119.5	C4—Ru1—C7	79.91 (17)
C13—C12—H12	119.5	O1—Ru1—C6	158.39 (14)
C12—C13—C14	121.5 (5)	O2—Ru1—C6	138.42 (14)
C12—C13—C18	118.9 (4)	C4—Ru1—C6	66.45 (16)
C14—C13—C18	119.6 (5)	C7—Ru1—C6	36.83 (16)
C15—C14—C13	122.2 (5)	O1—Ru1—C5	121.43 (13)
C15—C14—H14	118.9	O2—Ru1—C5	173.76 (13)
C13—C14—H14	118.9	C4—Ru1—C5	36.75 (15)
C14—C15—C16	118.5 (5)	C7—Ru1—C5	67.20 (16)
C14—C15—H15	120.7	C6—Ru1—C5	36.98 (16)
C16—C15—H15	120.7	O1—Ru1—C2	116.80 (15)
C17—C16—C15	121.6 (6)	O2—Ru1—C2	95.23 (13)
C17—C16—H16	119.2	C4—Ru1—C2	66.72 (19)
C15—C16—H16	119.2	C7—Ru1—C2	38.51 (18)
C16—C17—C18	121.7 (5)	C6—Ru1—C2	66.96 (15)
C16—C17—H17	119.1	C5—Ru1—C2	78.94 (16)
C18—C17—H17	119.1	O1—Ru1—C3	93.51 (13)
C9—C18—C17	124.7 (3)	O2—Ru1—C3	110.50 (13)
C9—C18—C13	119.0 (4)	C4—Ru1—C3	36.84 (18)
C17—C18—C13	116.3 (4)	C7—Ru1—C3	67.43 (18)
C20—C19—C24	110.0 (3)	C6—Ru1—C3	77.92 (14)
C20—C19—P1	111.8 (2)	C5—Ru1—C3	66.00 (15)
C24—C19—P1	116.4 (2)	C2—Ru1—C3	36.48 (18)
C20—C19—H19	106.0	O1—Ru1—P1	88.99 (6)
C24—C19—H19	106.0	O2—Ru1—P1	86.30 (6)
P1—C19—H19	106.0	C4—Ru1—P1	125.43 (13)
C19—C20—C21	110.3 (3)	C7—Ru1—P1	114.30 (13)
C19—C20—H20A	109.6	C6—Ru1—P1	93.47 (9)
C21—C20—H20A	109.6	C5—Ru1—P1	97.78 (11)
C19—C20—H20B	109.6	C2—Ru1—P1	151.84 (15)
C21—C20—H20B	109.6	C3—Ru1—P1	162.24 (13)
H20A—C20—H20B	108.1	O1—Ru1—C1	31.82 (10)
C20—C21—C22	110.2 (4)	O2—Ru1—C1	31.38 (10)
C20—C21—H21A	109.6	C4—Ru1—C1	120.25 (14)
C22—C21—H21A	109.6	C7—Ru1—C1	133.50 (15)
C20—C21—H21B	109.6	C6—Ru1—C1	169.46 (15)
C22—C21—H21B	109.6	C5—Ru1—C1	152.76 (14)
H21A—C21—H21B	108.1	C2—Ru1—C1	107.16 (13)
C23—C22—C21	111.5 (4)	C3—Ru1—C1	102.69 (12)
C23—C22—H22A	109.3	P1—Ru1—C1	88.63 (7)
C21—C22—H22A	109.3		

### Hydrogen-bond geometry (Å, º)

**Table:**

*D*—H···*A*	*D*—H	H···*A*	*D*···*A*	*D*—H···*A*
C6—H6···O3^i^	0.95	2.51	3.179 (5)	127
C29—H29*A*···Cl3^ii^	0.99	2.68	3.457 (6)	135
C31—H31···O3^iii^	1.00	2.06	3.004 (5)	156

Symmetry codes: (i) −*x*+1/2, *y*+1/2, *z*; (ii) *x*+1/2, −*y*+1/2, −*z*; (iii) *x*, *y*+1, *z*.
